# Adolescent Cyberviolence in South Korea: A Multi‐Year, National Population‐Based Study of Cyberviolence Prevalence (2017–2024)

**DOI:** 10.1002/bsl.70043

**Published:** 2026-02-09

**Authors:** Heng Choon (Oliver) Chan, Hong‐Jun Park, Evelyn Svingen

**Affiliations:** ^1^ Department of Social Policy, Sociology and Criminology School of Social Policy and Society University of Birmingham Birmingham UK

**Keywords:** adolescent, cyberviolence, cyberviolent behaviour, perpetration, South Korea, victimisation

## Abstract

With the rise of digital technology, adolescent cyberviolence has become a growing global concern in public health and criminal justice. This study used nationally representative data from South Korea (2017–2024) to examine the prevalence of eight types of cyberviolence (i.e., verbal abuse, defamation, stalking, sexual abuse, personal information leakage, bullying, extortion and coercion) among adolescents aged 10–18 years. It also analysed differences between sexes, age groups and time periods (i.e., before, during and after COVID‐19). The findings showed that male adolescents were generally more likely than females to be both victims and perpetrators of cyberviolence, except in the case of cyberstalking victimisation. The adolescents aged 13–15 reported higher rates of both victimisation and perpetration than their younger (aged 10–12) and older (aged 16–18) peers, except in the context of cyberbullying victimisation. Notably, cyberviolence rates were lower during the COVID‐19 pandemic (2020–2022) than the pre‐pandemic (2017–2019) and post‐pandemic (2023–2024) periods. This is arguably the first study to track cyberviolence among South Korean adolescents over an eight‐year period using data from over 56,000 participants. The results offer critical insights into patterns of online aggression among adolescents across sexes and age groups and through significant societal disruptions like the COVID‐19 pandemic.

## 1 Introduction

The Internet, social media (or commonly referred to as social networking sites in the South Korean context) and online communications have all changed the way people communicate and interact, globally. Information and communication technologies (ICTs) have become increasingly important in the lives of adolescents as a means to keep in contact with one another. Over the past decade, over 90% of European and American adolescents have become netizens (e.g., Lenhart et al. 2005; Livingstone et al. 2011; Taylor and Keeter 2010). In South Korea, 88% of primary school students in the 4th to 6th grades (aged 10–12 years) owned a personal mobile phone in 2020 (Korea Press Foundation 2020). The proliferation of ICTs lies at the centre of many challenges faced by today's societies, one such challenge being cyberviolence. ‘Cyberviolence’ generally refers to the use of technology, particularly the Internet and mobile devices, to harm, threaten, or harass individuals or groups of individuals (Mukred et al. 2024). Cyberviolence involves various types of harassment, violation of privacy, sexual exploitation, sexual abuse, and online threats, which can consist of bullying, sending threatening messages, stalking, releasing the victim's personal information (i.e., doxxing), money theft or fraud, and the distribution of sexually explicit images or videos without the consent of the individual depicted (i.e., revenge porn).

Although certain scholars have perceived ‘cyberviolence’ and ‘cyberbullying’ to be synonymous (e.g., Menesini et al. 2012), others have argued that cyberviolence is a broader concept than cyberbullying (e.g., Chun et al. 2024; Asad and Fatima 2024). S. M. Bae (2024) argued that the term ‘cyberbullying’ fails to encompass the wide range of emerging forms and features of digital violence, particularly given the emergence of a ‘born‐digital’ generation, the widespread use of smart devices, and the shift to online education due to the COVID‐19 pandemic. The nature of cyberviolence has evolved in diverse manners over recent years. In South Korea, the National Information Society Agency (National Information Society Agency 2025) defined ‘cyberviolence’ as ‘an action that harms others or gives discomfort and the feeling of anxiety to others verbally or via text message, videos, etc. in the cyberspace (i.e., internet, smartphone, etc.)’. The NIA identifies eight forms of cyberviolence: verbal/written, defamation, stalking, sexual/visual, invasion of privacy, exclusion, extortion, and coercion. This study adopted this operational definition and examined these varied forms of cyberviolence. Globally, the Broadband Commission for Digital Development (2015), as part of the International Telecommunication Union and the United Nations Educational, Scientific and Cultural Organization (UNESCO), classified the following six types of cyberviolence against girls and women: hacking, impersonation, surveillance/tracking, harassment/spamming, recruitment, and malicious distribution.

Prevalence rates for cyberviolence vary greatly across studies, due in part to methodological differences, such as sample characteristics, phenomenon conceptualisation, and assessment tools employed. It has been estimated that 20% of Germans, 40% of Americans and Egyptians, and up to 70% of Pakistanis have experienced cyberviolence victimisation (e.g., Al‐Nasrawi 2021; Duggan 2017; European Union Agency for Fundamental Rights 2014; Hassan et al. 2020; Staude‐Müller et al. 2012). Most empirical studies on cyberviolence conducted in South Korea have focussed on cyberbullying, which is arguably a subset of cyberviolence. The prevalence of cyberviolence perpetration and victimisation in South Korea remains unknown, particularly in the long term. The purpose of this study was to fill this gap.

Perception of violence is regarded to be a protective factor against actual violent behaviour (Niwako et al. 2009). Specifically, the literature has demonstrated that people who perceive violence or recognise problematic behaviour as acceptable and not a serious problem are more likely to engage in violent behaviour than others (S. Bae 2021a; Chan 2022; Lundgren and Amin 2015). Therefore, research on public perception can elucidate the extent to which the public is aware of and can recognise the nature and adverse consequences of cyberviolence incidents. More importantly, this line of research can help to address misconceptions that the public may hold about cyberviolence that contribute to a lack of public demand for necessary social and policy change. Given the potential high risk of adverse public health and criminal justice outcomes, studying this phenomenon in the context of South Korea is worthwhile, as it may provide a better understanding of such deviant behaviour in the nation. Furthermore, victimisation can lead to symptoms of strong psychological distress, including fear, depressed mood, and even self‐harm or suicide (Hinduja and Patchin 2010). Therefore, timely identification and prevention and intervention strategies are essential to prevent victimisation before perpetrators of cyberviolence escalate to more serious and chronic violent behaviours (e.g., physical violence).

Recently, research into adolescent cyberviolence (victimisation, perpetration, or both) has expanded, particularly in the wake of COVID‐19, but several gaps remain. For example, E. Lee et al. (2024a) analysed South Korean national survey data from 2019 to 2020 and found a decline in cyber victimisation during the pandemic, counter to the prevailing assumption that increased online time would lead to increased cyber harm. Younger age, more Internet use, exposure to violent media content, and being a perpetrator were all associated with higher risk of victimisation. K. Kim et al. (2024) identified latent profiles of cyber violence among South Korean adolescents, reporting involvement (as perpetrator, victim, or both) in about 41.6% of young people. The Korean National Cyber Violence Survey 2021 reported that cybervictimisation among adolescents occurred at a rate of 23.6% (Chun et al. 2024). Other studies, discussed in the following section, have examined exposure to violent content and media, the role of parental and school intervention, and perceptions of legal consequences; however, such studies have tended to be limited to a period of only one or 2 years, involve limited age ranges, or examine few forms of cyberviolence.

The relevant literature lacks a longer‐term, empirical study covering the years before, during, and after the COVID‐19 pandemic; including the full adolescent age span (e.g., 10–18 years); and encompassing all of the major roles involved in cyberviolence (i.e., victim, perpetrator, and overlapping roles), while also tracking demographics (e.g., sex, age) differences and indicators of prevention and awareness (e.g., help‐seeking, legal knowledge). This study addressed those gaps by using a large, nationally representative sample; a study period of eight consecutive years; the widest age range to date in the relevant research; and both victimisation and perpetration of cyberviolence, across multiple types of such violence. The present study was the first to use eight consecutive years (2017–2024) of nationally representative data to examine adolescents' experiences of cyberviolence in South Korea across the years before, during, and after COVID‐19. Furthermore, it covered the widest adolescent age range yet studied (10–18 years) and all eight forms of cyberviolence defined by the National Information Society Agency. In doing so, it provides the most comprehensive picture to date of the prevalence, trends and demographic differences in cyberviolence among South Korean adolescents and creates a robust evidence base to inform policy and intervention.

## 2 Types of Cyberviolence

### 2.1 Cyberbullying

Cyberbullying behaviour is broadly categorised into overt and relational forms of online aggression (Chan 2023; Vandebosch and Van Cleemput 2009; Willard 2007). Overt‐aggressive harassment is usually performed through electronic text, such as by sending threatening messages to the targeted victim. Conversely, relational‐aggressive behaviours may involve instances of exclusion (i.e., purposefully excluding the victim from partaking in online social chats or forums) and denigration (e.g., spreading rumours about the victim or publishing embarrassing photos or videos of the victim). Based on a review of 11 national studies (e.g., Canada, Malaysia, Singapore, South Korea, and the U.S.), Zhu et al. (2021) found that the global prevalence of cyberbullying incidents ranged from 14.6% to 52.2% for victimisation and from 6.3% to 32% for perpetration. Brochado et al. (2017), in their review of 159 empirical studies, found that lifetime prevalence rates of cyberviolence experience varied, with ranges of 4.9%–65% for victims, 1.2%–44.1% for perpetrators, and 5%–64.3% for participants who were both victims and perpetrators. The literature has demonstrated that cyberbullying perpetration and victimisation often peaks at 12–15 years of age (Slonje and Smith 2008; Walrave and Heirman 2011). Cyberbullying victimisation is more prevalent among primary school students than among middle and high school students; while cyberbullying perpetration is more commonly perpetrated by older adolescents than younger ones (Vismara et al. 2022). The literature has demonstrated that male adolescents are significantly more likely than female adolescents to engage in cyberbullying perpetration behaviour and to fall prey to cyberbullying victimisation (Ang and Goh 2010; Aricak et al. 2008; Li 2006; Slonje and Smith 2008; Wong et al. 2014).

### 2.2 Cyberstalking

‘Cyberstalking’ refers broadly to the use of the Internet and other technological devices (e.g., computers, smartphones, tablets) to repeatedly communicate with, monitor, or harass an individual in a threatening manner that can become intimidating or fear‐invoking (Bocij and McFarlane 2003; Reyns et al. 2012; Wilson et al. 2022). Prevalence rates have varied between empirical studies varied, in part due to discrepancies in operationalising cyberstalking (e.g., including or excluding fear as a defining criterion) and study duration. The lifetime prevalence of cyberstalking reported in these studies ranges from 6.5% to 46% for victimisation and from 4.9% to 50% for perpetration of cyberstalking (Berry and Bainbridge 2017; DeKeseredy et al. 2019; DeMatteo et al. 2017; Dreßing et al. 2014; Fansher and Randa 2019; Lyndon et al. 2011; Maran and Begotti 2019; Reyns et al. 2012; Strawhun et al. 2013). Among adolescents aged 10–17 years, the prevalence of cyberstalking has been reported as between 5% (Marcum et al. 2014) and 11% (Jones et al. 2013). In a cyberstalking victimisation study of 627 Portuguese adolescents aged 12–16 years, Pereira and Matos (2016) reported a prevalence rate of 61.9%, with a mean victim age of 14.26 years. Males are generally more likely than females to engage in cyberstalking perpetration behaviour, whereas females are cyberstalked more frequently than males (Dreßing et al. 2014; Maran and Begotti 2019; Pereira and Matos 2016).

### 2.3 Online Sexual Abuse

‘Online sexual abuse’, also commonly known as ‘digital or technology‐facilitated abuse’, refers to situations in which digital media, the Internet, and ICTs are used during sexual abuse or exploitation. It includes nonconsensual taking or sharing of images; threatening to share sexual pictures; unwanted sexual talk and questions; requests for sexual acts; and commercial sexual exploitation, involving sex talk, images, or other sexual activity (Finkelhor et al. 2022; Hamilton‐Giachritsis et al. 2020). The reported prevalence of online sexual abuse victimisation among adolescents varies from 5.8% to 51.3% (e.g., de et al. 2018; Jonsson et al. 2019; Mitchell et al. 2013; Montiel et al. 2016; Pedersen et al. 2023; Sklenarova et al. 2018). As with traditional, or offline, sexual abuse, certain studies have found that adolescent females are more likely than adolescent males to experience online sexual abuse (e.g., de et al. 2018; Jonsson et al. 2019; Mitchell et al. 2014; Montiel et al. 2016; Sklenarova et al. 2018), while other studies have failed to find any differences in this kind of victimisation between the sexes (e.g., Pedersen et al. 2023; Toomey and Russell 2016). In terms of age, Montiel et al. (2016) found that older adolescents (16–17 years old) reported more online sexual victimisation (47.1%) than did younger adolescents (i.e., 41.4% in adolescents aged 14–15 years and 30.1% in those aged 12–13 years) in most types of online sexual abuse (i.e., sexual pressure, sexual grooming, and exposing victims to unwanted sexual content).

### 2.4 Other Types of Cyberviolence

In addition to cyberbullying, cyberstalking, and online sexual abuse, other, less commonly researched types of cyberviolence are relevant to this study, including cyber extortion, which is an act of conditional speech intended to influence or gain compliance of an individual or a group of individuals (Spitzberg and Gawron 2016). Extortionate and threatening communication such as intimidation, isolation, and control can be instrumental in obtaining financial or psychological control over victims online (Eisenberg 2015; Grabosky et al. 2001). ‘Coercive control’ refers to the ongoing application of abusive tactics designed to limit a victim's decision‐making ability by denying their liberty, autonomy, and equality and thereby establishing a power imbalance between the perpetrator and the victim, which is exploited by the former (Stark 2007). Coercive control is frequently studied in the context of domestic and intimate partner violence (Karystianis et al. 2024; Smith et al. 2024). Cyber coercion is the use of such abusive tactics in a cyber setting through the use of communicative text or images. Other, less studied cyberviolence behaviours include defamatory acts (i.e., insulting, harassing, or slandering the victim), verbal abuse, and leaking personal information through electronic means (Balci and Salah 2015; Prayitno and Bawono 2023).

## 3 The Cyber Violence Survey in South Korea

South Korea is regarded as one of the most technologically advanced and highly connected countries, with an Internet usage rate that has steadily increased over the past decade, from 88% in 2014 to 97% in 2023, which is the highest rate in Asia (World Bank Open Data 2025). Taking advantage of the country's fast and relatively cheap broadband connectivity (Falcon 2020), South Korean adolescents tend to possess personal mobile devices, especially smartphones, at a relatively young age (Odgers and Jensen 2020). The increase in online activity witnessed during the COVID‐19 pandemic continued after the pandemic, especially for leisure (e.g., watching cultural content via online streaming platforms such as YouTube and Netflix), which increased from less than 30% of the population consuming such media in 2019 to over 50% in 2022 (Statistics Korea 2023). Household Internet access, household computer ownership, and individual smartphone ownership rates in South Korea reached 99.9%, 77.2%, and 97.3%, respectively, in 2024 (Ministry of Science and ICT 2025). These phenomena have also increased the occurrence of cyberviolence incidents and other forms of cybercrime in South Korea.

The annually administered Cyber Violence Survey was initiated in 2013 by the Korea Communications Commission (KCC), a presidentially sanctioned regulatory body. The KCC is responsible for regulating broadcasting and communication services, protecting users, and addressing related issues to maintain the independence of broadcasting services in South Korea. Its nationally representative survey aimed to address concerns about cyberviolence targeting children and adolescents by developing a comprehensive understanding of prevailing issues in South Korea. The KCC entrusted the survey administration and results generation to the National Information Society Agency, which was established in 1987 under the Ministry of Science and ICT, and the Ministry of the Interior and Security.

To the authors' knowledge, a total of 13 recently published studies have analysed the results of the Cyber Violence Survey, examining variables including types of deviant online behaviours (e.g., perceptions and experiences of cyber‐victimisation, cyberbullying perpetration, online sex crimes, and cyberviolence) and exposure to risky and violent online media (see, e.g., S. Bae 2021a, 2021b; Choi et al. 2022; Jo 2025; Jun 2020; D. Kim and Lee 2025; H. Lee and Jun 2024; E. Lee and Lee 2024; E. Lee et al. 2024a, 2024b; Paek et al. 2022, 2023; Shin and Choi 2021). However, most of these studies explored only a single year of data and ended by 2022, while a few examined two or three years of data, and only one study analysed five years of data (2018–2022; E. Lee et al. 2024b). Hence, the trends in perceptions and experiences of cyberviolence among adolescents in South Korea over longer periods remain unknown.

Seven of these studies analysed a single year of survey data (i.e., S. Bae 2021a, 2021b [2019 survey]; Jo 2025 [2022 survey]; D. Kim and Lee 2025 [2020 survey]; H. Lee and Jun 2024 [2021 survey]; Paek et al. 2022, 2023 [2020 survey]). Analysing the data regarding 3202 adolescents' 2019 survey responses, S. Bae (2021a) reported that cyber offending was positively related to exposure to violent online media but negatively correlated with the perception of cyberviolence (e.g., awareness and personal judgement of the phenomenon). Bae's study also found that the effect of exposure to violent online media on cyber offending was moderated by the perception of cyberviolence. In another study, with a sample of 4779 adolescents, S. Bae (2021b) observed that the male adolescents reported significantly more cyberbullying perpetration than the female adolescents and that the frequency of cyberbullying perpetration among the adolescents decreased as they aged (from elementary school ages to middle and high school ages). Although cyberbullying victimisation was positively associated with cyberbullying perpetration, the perception of cyberbullying was negatively related to cyberbullying perpetration.

Using the 2020 data from 4958 adolescents' survey responses, Paek et al. (2022) studied the predictors of cyberbullying victimisation during the COVID‐19 pandemic period. In addition to male adolescents' higher risk of becoming victims of cyberbullying than female adolescents, they found that a longer duration of Internet use and online activities that involved interaction with other users increased their likelihood of falling prey to victimisation, while parental guardianship provided partial protection, reducing non‐violent victimisation (e.g., leakage of personal information). In another study examining the moderating role of adolescents' exposure to harmful online content on their cyberbullying perpetration during the COVID‐19 pandemic period, using the 2020 survey data, Paek et al. (2023) confirmed this relationship, finding that those who had encountered risky content were more likely to cyberbully others than those without such an experience. They also found that supportive parenting had varying effects to the level of exposure to risky content. For instance, an increased likelihood of cyberbullying perpetration was found in a portion of adolescents who had a high level of exposure to harmful online content compared to their less exposed counterparts. Similarly analysing the 2020 survey data of 4958 adolescent respondents, D. Kim and Lee (2025) explored the relationship between cyberbullying perception, victimisation, and perpetration and the adolescents' social support and amount of Internet use. In addition to a positive correlation between cyberbullying victimisation and perpetration, they found that higher perceptions of cybercrime (e.g., greater awareness of cyberbullying) were associated with a lower likelihood of cyberbullying victimisation, while adolescents who spent more time engaged in online activities displayed an increased chance of being victimised. In contrast, adolescents who received greater support from parents and friends and preventive education at school had lower chances of experiencing victimisation.

To explore the relationships between exposure to risky online content, moral disengagement, media literacy, and cyberaggression in adolescents, H. Lee and Jun (2024) analysed the 2021 survey responses of 3002 adolescents aged 13–15 years. They found a positive correlation between exposure to risky online content and moral disengagement among adolescents, which was mitigated by higher levels of media literacy. Moral disengagement related to cyberaggression increased the likelihood of adolescents engaging in aggressive online behaviour, whereas a high level of media literacy reduced both their cyberaggression and moral disengagement. Jo (2025) studied a sample of 1999 cyberbullying perpetrators identified in the 2022 survey data to explore the effects of prejudice, exposure to harmful content, digital sex crime, and cyberbullying education on cyberbullying behaviours, using the partial least squares structural equation modelling approach. The findings indicated that prejudice (i.e., biased or unfavourable attitudes towards individuals based on factors such as race, religion, and sexual orientation), exposure to harmful content, and digital sex crimes were positively associated with cyberbullying, while cyberbullying education was positively correlated with online harassment perception (i.e., recognising and rationalising online harassment as aggressive and harmful behaviour). However, a respondent's act of disclosing their involvement in cyberbullying was also positively correlated to both showing remorse and rationalisation among adolescents, which underscored the complex interplay between individual attitudes, online experiences and educational interventions in fostering cyberbullying behaviours.

Four of the relevant studies examined survey data over 2‐year periods (i.e., Choi et al. 2022 [2019 and 2020 surveys]; E. Lee and Lee 2024 [2021 and 2022 surveys]; E. Lee et al. 2024a [2019 and 2020 surveys]; Shin and Choi 2021 [2019 and 2020 surveys]). Shin and Choi (2021) examined the trends of cyberbullying before and after the COVID‐19 pandemic, with a sample of 9737 adolescents, and found that the rate of cyberbullying experience, either perpetration or victimisation, was lower in 2020 (22.8%) than in 2019 (26.9%). They found a sharp decline in cyberbullying perpetration (9.5% in 2020, 18% in 2019). These decreasing rates in cyberbullying experience between 2019 and 2020 were similar for the adolescents in elementary, middle, and high schools (0.9%, 13.8%, and 11.3% declines, respectively). Although some differences were observed between the two studied years, being male, exposure to harmful online content, having more friends perpetrating cyberbullying, lower levels of parent–child interaction, and witnessing cyberbullying were significant predictors of cyberbullying perpetration in both 2019 and 2020. Choi et al. (2022) analysed the 2019 and 2020 survey responses of 3315 elementary school students (aged 10–12 years) to explore the factors influencing the experience of cyberbullying during the COVID‐19 pandemic. Regardless of the pandemic, being male, having more friends who perpetrated cyberbullying, exposure to harmful online content, and witnessing cyberbullying were significantly associated with cyberbullying perpetration, while higher levels of parent–child interaction, school involvement, and awareness of cyberbullying issues were significant predictors of cyberbullying perpetration in 2020 (after COVID‐19 became prevalent).

Similarly, examining the 2019 and 2020 survey data, E. Lee et al. (2024a) reported that cyberviolence perpetration and victimisation were positively correlated, and younger adolescents, those who spent more time online, and those who had been exposed to violent media were significantly more susceptible to victimisation than the other respondents. Examining a total of 18,709 adolescents and using the 2021 and 2022 data, E. Lee and Lee (2024) studied the relationship between cyberviolence and cyber‐sex crimes and found that adolescents who were exposed to violent media and cyber victimisation were at an increased risk of encountering online sexual content. Female and older adolescents appeared to be more likely to witness cyber sex crimes than their counterparts (i.e., male and younger adolescents).

Only two of the studies analysed survey data over longer periods, one covering three years (Jun 2020, 2017–2019 surveys) and one covering five years (E. Lee et al. 2024b, 2018–2022 surveys). With a total sample of 13,941 adolescents, Jun (2020) reported that the most frequent type of cyberbullying perpetration and victimisation was one‐on‐one verbal abuse (i.e., a single perpetrator and a single victim), and instant messaging constituted the most frequently used means of cyberbullying. The adolescents who actively interacted with parents and reliable friends were less likely to experience cyberbullying. Analysing five years of survey data with a sample of 33,108 adolescents, E. Lee et al. (2024b) found that the adolescents who were more exposed to violent media content, who were younger, and who themselves engaged in cyberviolence were more likely to witness cyberviolence (as an innocent bystander). Conversely, those with less exposure to violent media content and who were aware of viable responses when witnessing cyberviolence were more likely to adopt good citizenship behaviours (e.g., advocating ethical behaviours, ensuring online safety).

### 3.1 The Present Study

Against this background, the importance of the present study is twofold. First, it is the first empirical study of the prevalence of cyberviolence in South Korean adolescents to span an eight‐year period starting prior to the COVID‐19 pandemic and ending after the pandemic, using nationally representative data. This study also explored differences between sexes and age groups, as earlier works indicated that males and females and those in different age groups may differ in their experiences of different types of cyberviolence (e.g., Chan 2023, 2024; Chan and Wong 2015, 2019). Thus, the present study is essential in advancing our understanding of cyberviolence in adolescents. The study also provides the first nationally representative evidence of sex and age‐group differences across all eight officially defined types of cyberviolence in South Korea, using the widest adolescent age range (10–18 years) analysed to date. Importantly, the findings of this study may shed light on how to improve cyberviolence prevention and intervention strategies for adolescents not only in South Korea but globally.

Drawing from the literature, the following research hypotheses were proposed.

Hypothesis 1 There are differences in the experience of cyberviolence between the sexes. Specifically, females are more likely than males to experience victimisation, while males perpetrate more cyberviolence than females.

Hypothesis 2 There are differences in the experience of cyberviolence between age groups. Specifically, younger adolescents experience more cyberviolence victimisation than older adolescents, while older adolescents are more likely to perpetrate cyberviolence than younger adolescents.

Hypothesis 3 Given the increased amount of time adolescents spend engaging in online activities, in part due to the widespread adoption of remote learning during the COVID‐19 pandemic years (2020–2022), cyberviolence incidents occurred more frequently during these years than in the pre‐pandemic (2017–2019) and post‐pandemic (2023–2024) periods.

## 4 Method

### 4.1 Data and Procedure

In this study, the Cyber Violence Survey data from an eight‐year period (2017–2024) were requested and analysed. Because the survey method changed in 2017, data before 2017 were not included in this study. It is worth noting that the surveys were conducted in different ways over the years; specifically, the responses were collected in group surveys administered during school visits, via postal surveys, and via online surveys. The data were originally collected using a two‐stage, stratified cluster sampling method. Schools were first stratified by region (17 cities and provinces across South Korea) and school type, then selected through systematic sampling from the School Statistics database managed by the Korea Educational Development Institute. One class was randomly chosen from each selected school, and all students in that class were surveyed. This stratified sampling approach involved organising homogenous subgroups from the population and extracting samples according to specific representativeness. The homogeneity of these groups aimed to reduce sampling errors and ensured the representativeness of related variables. The sample consisted of South Korean students from the 4^th^ grade (elementary school) through the 12th grade (high school); that is, the last three years of elementary school, all three years of middle school, and all three years of high school. The majority of the respondents were aged 10–18 years.

During this eight‐year period, a total of 56,306 participants were surveyed (4500 [8.0% of this study's sample] in 2017, 4662 [8.3%] in 2018, 4779 [8.5%] in 2019, 4779 [8.8%] in 2020, 9017 [16.0%] in 2021, 9693 [17.2%] in 2022, 9218 [16.4%] in 2023, and 9479 [16.8%] in 2024), with similar proportions of males (52%) and females (48%). The mean age of the participants was 13.90 years (SD = 2.54). The participants' ages were also distributed relatively evenly between early (34.1%, 10–12 years old), middle (33.1%, 13–15 years old), and late (32.8%, 16–18 years old) adolescence. Ethical approval was not required for the present study, which used publicly available data without personal identifiers.

### 4.2 Measures

In this study, nine measures were used to analyse the participants' daily online routines, experience witnessing cyberviolence incidents, perception of various types of cyberviolence as problematic, experience with cyberviolence victimisation and perpetration, perception of education as an important and helpful preventive measure, knowledge about means to seek support and to report cyberviolence incidents, and knowledge about the legal consequences of perpetrating cyberviolence. These measures are described in detail below.

*Daily online routines*. The participants' daily online routines were measured by their average daily Internet usage time (for personal and academic purposes) over the previous 12 months, with the following answer options: ‘Less than 1 hour’, ‘1 hour to less than 2 hours’, ‘2 hours to less than 3 hours’, ‘3 hours to less than 5 hours’, and 5 hours’ or more. This measure was only included in the surveys conducted between 2021 and 2024, inclusive.

*Witnessing cyberviolence*. The participants were asked to report if they had witnessed the following types of cyberviolence in the previous year: perpetration, victimisation, both perpetration and victimisation, and no experience in witnessing any cyberviolence. The surveys issued in 2017–2020 provided only ‘Witnessed cyberviolence’ and ‘Have not witnessed cyberviolence’ as response options.

*Cyberviolence types*. Eight items were used to measure types of cyberviolence: (1) ‘Swore at someone or hurt someone's feelings’ (cyber verbal abuse); (2) ‘Spread false or exaggerated stories about another person’ (cyber defamation); (3) ‘Sent emails or messages to another person despite their desire not to receive them or continuously visited someone's blog or social networking site (e.g., Instagram, Facebook, X, TikTok) to post unwanted text or photos’ (cyberstalking); (4) ‘Sent sexually suggestive text, photos, or videos via computer or smartphone knowing the other person would dislike it’ (online sexual abuse); (5) ‘Spread someone's personal information such as name, address, school, and photos’ (leakage of personal information); (6) ‘Prevented someone from leaving, teased or swore at them, or excluded them from conversations in an Internet chatroom or smartphone messaging app (e.g., KakaoTalk, WhatsApp, Line, Facebook Messenger, Instagram Direct Message, Telegram)’ (cyberbullying); (7) ‘Forcibly took someone's cybergame money, smartphone data, game items, or other items of a similar nature’ (cyber extortion); and (8) ‘Forced someone to do or say something they did not want to or made them run errands via Internet communication’ (cyber coercion). Three items were used to measure the participants' perceptions and experiences (perpetration and victimisation) of cyberviolence: (1) ‘Do you think the following behaviours in the online space are problematic?’ to measure perception of cyberviolence; (2) ‘In the past year, have you experienced the following behaviours in the online space?’ to measure experience of cyberviolence victimisation; and (3) ‘In the past year, have you engaged in the following behaviours in the online space?’ to measure cyberviolence perpetration. These were phrased as yes/no questions (0 = *no*, 1 = *yes*). The participants' perceptions of cyberviolence were only measured in 2018–2022.

*Perception of and knowledge about prevention and intervention*. Four items were used to assess the participants' perceptions and knowledge concerning preventing their victimisation and coping with cyberviolence in general. The items were: (1) ‘Education related to cyberviolence prevention and response is important and necessary’, (2) ‘Education related to cyberviolence prevention and response is helpful’, (3) ‘Are you aware of official websites and phone numbers for counselling or reporting cyberviolence incidents?’, and (4) ‘Are you aware of the severity or nature of the legal consequences of engaging in cyberviolence?’ These were phrased as true/false statements and yes/no questions (0 = *no/false*, 1 = *yes/true*). The participants' perceptions of education as an important and helpful preventive measure were only measured beginning in 2021.

### 4.3 Data Analysis

Cross‐tabular (i.e., chi‐squared, *χ* ^2^) analyses were performed using SPSS 29.0 to explore the significance of differences in the participants' cyberviolence perceptions and experiences based on their sex, their age, and year of data collection. This analytic method was used primarily due to the categorical nature of the variables of interest. Given that statistically significant relationships are more likely to occur in a large dataset such as the one in this study, measures of association (i.e., Phi and Cramér's *V* coefficients) were used to interpret the strength of the relationship and, most importantly, to detect meaningful patterns. A relationship of 1.00 indicates a perfect relationship. Adopting Cohen's standard for the interpretation of chi‐square effect sizes, for 2 × 2 tables with one degree of freedom (e.g., the participants' perceptions and experiences of the types of cyberviolence behaviour), Phi values of 0.29 and below were considered weak, those between 0.30 and 0.49 were considered moderate, and those of 0.50 and above were regarded as strong (Gravetter et al. 2017). In chi‐square analyses of 2 × 3 (e.g., the participants' age categories and school levels), 2 × 4 (e.g., the participants' experience of cyberviolence), and 2 × 5 (e.g., the participants' daily online routines) matrices, Cramér's V values of 0.16 and below were regarded as weak effects, those between 0.17 and 0.28 were considered moderate, and those of 0.29 and above were considered strong effects (Gravetter et al. 2017). When the degrees of freedom exceeded one (e.g., a 2 × 3 matrix), the Bonferroni correction method was applied when comparing the column proportions. The significance level was set at 0.05. No data imputation was performed. The raw data were used to best reflect the actual phenomenon. The authors take responsibility for the integrity of the data and the accuracy of the data analyses and have made every effort to avoid inflating statistically significant results.

## 5 Results

### 5.1 General Results

The relevant survey response data are shown in Table 1 and are summarised as follows (multiple options were allowed for cyberviolence victimisation and perpetration, for example, a participant can experience more than one type of cyberviolence during the previous year). Although nearly two thirds of the adolescents (74.2%) engaged in online activities for two to 5 hours daily, a large majority of them (89.9%) recognised various types of online behaviour as problematic, from online verbal abusive behaviour (92.8%) to the online leakage of personal information (93.7%). However, most of the adolescents (91%) reported not having witnessed any type of cyberviolence in the previous year. In this study, only about two‐fifths of the adolescents reported to have fallen victim to any type of cyberviolence (17.9%, ranging from 1.3% for cyber extortion to 14.8% for cyber verbal abuse) or committed cyberviolence (18%, ranging from 1.3% for cyber extortion to 16.2% for cyber verbal abuse). Incidents of verbal abuse in the digital world were the most common type of cyberviolence in South Korea, according to the survey results. Even though a large majority of the adolescents acknowledged the importance of preventive education (92.6%) and the helpfulness of such education in preventing the occurrence of cyberviolence (86.1%), not many of them were aware of either the resources available for victims through official channels (e.g., information and counselling services provided by governmental or social service agencies) (52.2%) or the legal consequences of cyberviolence perpetration (63.3%).

**TABLE 1:** Male and female adolescents' demographic characteristics and cyberviolence perception and experience (*N* = 56,306).

Characteristics	All sample (*N* = 56,306)		Male (*n* = 29,179)		Female (*n* = 27,127)		Group differences	
	*N*	%	*n*	%	*n*	%	*χ* ^2^ (df)	Phi/Cramer's *V*
Age (*n* = 51,348)	13.90 (SD = 2.54)		13.98 (SD = 2.53)		13.91 (SD = 2.54)		*t* = 7.89***	
Age categories							76.57 (2)	0.04***
(*n* = 51,348; male = 26,611, female = 24,737)								
Early adolescence (10–12 years)	17,514	34.1	8648^a^	32.5	8,866^b^	35.8		
Middle adolescence (13–15 years)	16,998	33.1	8860	33.3	8138	32.9		
Late adolescence (16–18 years)	16,836	32.8	9103^a^	34.2	7733^b^	31.3		
School level							78.66 (2)	0.04***
(*N* = 56,306; male = 29,179, female = 27,127)								
Primary school (grade 4–6)	19,243	34.1	9,521^a^	32.6	9,722^b^	35.8		
Middle school (grade 7–9)	18,618	33.1	9693	33.2	8925	32.9		
Highschool (grade 10–12)	18,445	32.8	9,965^a^	34.2	8,480^b^	31.3		
Daily online routine							190.13 (4)	0.07***
(*n* = 37,407; male = 18,995, female = 18,412)								
Less than an hour	2604	7.0	1,429^a^	7.5	1,175^b^	6.4		
1 hour to less than 2 hours	7037	18.8	3,804^a^	20.0	3,233^b^	17.6		
2 hours to less than 3 hours	9449	25.3	5,095^a^	26.8	4,354^b^	23.6		
3 hours to less than 5 hours	9961	26.6	4,853^a^	25.5	5,108^b^	27.7		
5 hours or more	8356	22.3	3,814^a^	20.1	4,542^b^	24.7		
Recognised as problematic behaviour	29,762	89.9	14,902	87.4	14,860	92.6	247.89 (1)	−0.09***
(*n* = 33,109; male = 17,058, female = 16,051)								
Cyber verbal abuse	30,737	92.8	15,514	90.9	15,223	94.8	188.43 (1)	−0.08***
Cyber defamation	30,944	93.5	15,699	92.0	15,245	95.0	117.39 (1)	−0.06***
Cyberstalking	30,842	93.2	15,614	91.5	15,228	94.9	144.45 (1)	−0.07***
Online sexual abuse	30,964	93.5	15,701	92.0	15,263	95.1	126.62 (1)	−0.06***
Personal information leakage	31,009	93.7	15,732	92.2	15,277	95.2	121.26 (1)	−0.06***
Cyberbullying	30,919	93.4	15,664	91.8	15,255	95.0	138.20 (1)	−0.07***
Cyber extortion	30,843	93.2	15,640	91.7	15,203	94.7	119.06 (1)	−0.06***
Cyber coercion	30,939	93.4	15,702	92.1	15,237	94.9	111.84 (1)	−0.06***
Witnessed cyberviolence							24.27 (3)	0.02***
(*N* = 56,306; male = 29,179, female = 27,127)								
Perpetration	387	0.7	226^a^	0.8	161^b^	0.6		
Victimisation	1108	2.0	506^a^	1.7	602^b^	2.2		
Perpetration and victimisation	3561	6.3	1871	6.4	1690	6.2		
Have not witnessed	51,250	91.0	26,576	91.1	24,674	91.0		
Cyberviolence victimisation	10,058	17.9	6035	20.7	4023	14.8	328.17 (1)	−0.08***
(*N* = 56,306; male = 29,179, female = 27,127)								
Cyber verbal abuse	8333	14.8	5165	17.7	3168	11.7	404.40 (1)	−0.09***
Cyber defamation	3303	5.9	1869	6.4	1434	5.3	31.88 (1)	−0.02***
Cyberstalking	1891	3.4	918	3.1	973	3.6	8.41 (1)	0.01**
Online sexual abuse	1358	2.4	795	2.7	563	2.1	25.17 (1)	−0.02***
Personal information leakage	1228	2.2	751	2.6	477	1.8	43.81 (1)	−0.03***
Cyberbullying	1038	1.8	655	2.2	383	1.4	53.89 (1)	−0.03***
Cyber extortion	731	1.3	529	1.8	202	0.7	125.20 (1)	−0.05***
Cyber coercion	774	1.4	482	1.7	292	1.1	34.34 (1)	−0.03***
Cyberviolence perpetration	10,141	18.0	6560	22.5	3581	13.2	820.03 (1)	−0.12***
(*N* = 56,306; male = 29,179, female = 27,127)								
Cyber verbal abuse	9121	16.2	5935	20.3	3186	11.7	765.06 (1)	−0.12***
Cyber defamation	2419	4.3	1580	5.4	839	3.1	184.34 (1)	−0.06***
Cyberstalking	1068	1.9	731	2.5	337	1.2	120.50 (1)	−0.05***
Online sexual abuse	855	1.5	619	2.1	236	0.9	147.21 (1)	−0.05***
Personal information leakage	1264	2.2	845	2.9	419	1.5	116.98 (1)	−0.05***
Cyberbullying	1127	2.0	769	2.6	358	1.3	124.07 (1)	−0.05***
Cyber extortion	717	1.3	538	1.8	179	0.7	156.74 (1)	−0.05***
Cyber coercion	769	1.4	526	1.8	243	0.9	85.83 (1)	−0.04***
Preventive education is important	34,651	92.6	17,069	89.9	17,582	95.5	434.47 (1)	0.11***
(*n* = 37,407; male = 18,995, female = 18,412)								
Preventive education provided is helpful	28,768	86.1	14,228	85.3	14,540	86.8	16.35 (1)	0.02***
(*n* = 33,427; male = 16,681, female = 16,746)								
Aware of official help provided	29,369	52.2	15,517	53.2	13,852	51.1	25.20 (1)	0.02***
(*N* = 56,306; male = 29,179, female = 27,127)								
Aware of legal consequences		35,654	63.3	18,734	64.2	16,920	62.4	20.28 (1)
(*N* = 56,306; male = 29,179, female = 27,127)								

*Note:* Subscripts (a, b) indicate significant differences.

*** *p* < 0.001.

** *p* < 0.01.

### 5.2 Sex Differences in Perception and Prevalence of Cyberviolence Experience

Table 1 presents the differences between male and female adolescents' cyberviolence perceptions and experiences in the 12‐month period prior to the survey. Significant differences were found between the sexes. Specifically, relative to male adolescents, female adolescents spent more time in a day on online activities, and this difference was significant (52.4% vs. 45.6% spent at least three or more hours a day; *χ* ^2^ = 190.13, Cramér's *V* = 0.07, *p* < 0.001). A significantly larger proportion of female adolescents than male adolescents were able to identify types of cyberviolence as problematic (92.6% vs. 89.9%; *χ* ^2^ = 247.89, Phi = −0.09, *p* < 0.001), across types of such behaviour (94.7%–95.2% vs. 90.9%–92.2%). The most commonly recognised type of problematic online behaviour was leakage of personal information (95.2% in females and 92.2% in males). Although most of the male (91%) and female (91.1%) adolescents had not witnessed any cyberviolence during the previous 12 months, significantly more of the males than the females had witnessed perpetration of cyberviolence (0.8% vs. 0.6%), and 2.2% of the female adolescents had witnessed victimisation of cyberviolence (vs. 1.7% of the males) (*χ* ^2^ = 24.27, Cramér's *V* = 0.02, *p* < 0.001).

In terms of personally experiencing cyberviolence, the male adolescents were significantly more likely than the female adolescents to have experienced both victimisation (20.7% vs. 14.8%; *χ* ^2^ = 328.17, Phi = −0.08, *p* < 0.001) and perpetration (22.5% vs. 13.2%; *χ* ^2^ = 820.03, Phi = −0.12, *p* < 0.001) across most types of cyberviolence. For both the male and female adolescents, the most frequently experienced type of cyberviolence as either victim or perpetrator was online verbal abuse (17.7% males and 11.7% females as victim, and 20.3% males and 11.7% females as perpetrator). Both of these differences were significant. The female adolescents were significantly more likely than the male adolescents to experience cyberstalking only as the victim (3.6% vs. 3.1%). Cyber extortion and cyber coercion were experienced the least by both the male and female adolescents.

In terms of perceptions about the prevention of and response to cyberviolence, significantly more of the female than the male adolescents recognised the importance of preventive education (95.5% vs. 89.9%; *χ* ^2^ = 434.47, Phi = 0.11, *p* < 0.001) and acknowledged that the information provided concerning how to respond to cyberviolence was helpful (86.8% vs. 85.3%; *χ* ^2^ = 16.35, Phi = 0.02, *p* < 0.001). However, relative to the female adolescents, the male adolescents were significantly more likely to know where to ask for help or obtain resources to cope with their own victimisation (53.2% vs. 51.1%; *χ* ^2^ = 25.20, Phi = 0.02, *p* < 0.001) and to understand the legal consequences of perpetrating cyberviolence (64.2% vs. 62.4%; *χ* ^2^ = 20.28, Phi = 0.02, *p* < 0.001). The strength of association in all of the differences between the male and female adolescents was weak, with Phi and Cramér's *V* values ranging from 0.01 to 0.12.

### 5.3 Age Differences in Perception of Cyberviolence and Prevalence of Cyberviolence Experience

The results concerning the respondents' perceptions of cyberviolence and the prevalence of their cyberviolence experience by age group (i.e., young [aged 10–12 years], middle [age 13–15 years], and older [aged 16–18 years] adolescents) are shown in Table 2. A significant, upward trend in daily online activity among young, middle, and older adolescents was observed (*χ* ^2^ = 3450.94, *p* < 0.001). The young adolescents were more likely to go online between one and 3 hours daily (54.3%), while most of the middle adolescents surfed the Internet between two and 5 hours daily (57.6%) and most of the older adolescents engaged in online activities for three or more hours daily (59.5%). This association approached the level of moderate (Cramér's *V* = 0.22). Similarly, an increase in the recognition of problematic online behaviour, in general, was observed as the adolescents aged. Specifically, 86.4% of the young adolescents, 90.5% of the middle adolescents, and 91.4% of the older adolescents recognized such behaviour as problematic, representing a significant group difference (*χ* ^2^ = 143.45, Cramér's *V* = 0.07, *p* < 0.001). This statistical pattern was evident across all of the studied types of cyberviolence. In contrast, the younger adolescents reported having witnessed significantly more cyberviolence incidents than their older counterparts (11% of young adolescents, 9.2% of middle adolescents, and 6.6% of older adolescents; *χ* ^2^ = 352.15, Cramér's *V* = 0.06, *p* < 0.001). However, the strength of association in these group differences was weak (0.06–0.09).

**TABLE 2:** Young, middle and older adolescents' cyberviolence perception and experience (*n* = 51,348).

Characteristics	Young adolescents(n = 17,514)		Middle adolescents(n = 16,998)		Older adolescents(n = 16,836)		Group differences	
	*N*	%	*n*	%	*n*	%	*χ* ^2^ (df)	Phi/Cramer's *V*
Daily online routine							3450.94 (8)	0.22***
(*n* = 37,407; young adolescent = 12,949, middle adolescent = 12,374, older adolescent = 12,084)								
Less than an hour	1,728^a^	13.3	426^b^	3.4	450^b^	3.7		
1 hour to less than 2 hours	3,731^a^	28.8	1,725^b^	13.9	1,581^b^	13.1		
2 hours to less than 3 hours	3,301^a^	25.5	3,286^a^	26.6	2,862^b^	23.7		
3 hours to less than 5 hours	2,491^a^	19.2	3,836^b^	31.0	3,634^b^	30.1		
5 hours or more	1,698^a^	13.1	3,101^b^	25.1	3,557^c^	29.4		
Recognised problematic behaviour	8,213^a^	86.4	8,450^b^	90.5	8,501^b^	91.4	143.45 (2)	0.07***
(*n* = 28,151; young adolescent = 9,511, middle adolescent = 9,337, older adolescent = 9303)								
Cyber verbal abuse	8,545^a^	89.8	8,734^b^	93.5	8,775^b^	94.3	156.88 (2)	0.08***
Cyber defamation	8,563^a^	90.0	8,806^b^	94.3	8,831^b^	94.9	208.11 (2)	0.09***
Cyberstalking	8,531^a^	89.7	8,766^b^	93.9	8,827^c^	94.9	213.99 (2)	0.09***
Online sexual abuse	8,586^a^	90.3	8,814^b^	94.4	8,809^b^	94.7	179.36 (2)	0.08***
Personal information leakage	8,567^a^	90.1	8,830^b^	94.6	8,856^b^	95.2	234.37 (2)	0.09***
Cyberbullying	8,558^a^	90.0	8,780^b^	94.0	8,836^c^	95.0	203.99 (2)	0.09***
Cyber extortion	8,521^a^	89.6	8,770^b^	93.9	8,825^c^	94.9	222.68 (2)	0.09***
Cyber coercion	8,550^a^	89.9	8,810^b^	94.4	8,826^b^	94.9	217.80 (2)	0.09***
Witnessed cyberviolence							352.15 (6)	0.06***
(*n* = 51,348; young adolescent = 17,514, middle adolescent = 16,998, older adolescent = 16,836)								
Perpetration	207^a^	1.2	110^b^	0.6	70^c^	0.4		
Victimisation	600^a^	3.4	326^b^	1.9	182^c^	1.1		
Perpetration and victimisation	1,128^a^	6.4	1,120^a^	6.6	860^b^	5.1		
Have not witnessed	15,579^a^	89.0	15,442^b^	90.8	15,724^c^	93.4		
Cyberviolence victimisation	3,095^a^	17.7	3,530^b^	20.8	2,457^c^	14.6	221.42 (2)	0.07***
(*n* = 51,348; young adolescent = 17,514, middle adolescent = 16,998, older adolescent = 16,836)								
Cyber verbal abuse	2,469^a^	14.1	3,018^b^	17.8	2,042^c^	12.1	220.77 (2)	0.07***
Cyber defamation	970^a^	5.5	1,244^b^	7.3	764^c^	4.5	25.55 (2)	0.02***
Cyberstalking	544^a^	3.1	656^b^	3.9	495^a^	2.9	19.43 (2)	0.02***
Online sexual abuse	336^a^	1.9	495^b^	2.9	385^a^	2.3	37.55 (2)	0.03***
Personal information leakage	283^a^	1.6	469^b^	2.8	322^a^	1.9	58.98 (2)	0.03***
Cyberbullying	367^a^	2.1	306^a^	1.8	218^b^	1.3	32.90 (2)	0.03***
Cyber extortion	210^a,b^	1.2	228^b^	1.3	174^a^	1.0	6.82 (2)	0.01**
Cyber coercion	242^a,b^	1.4	253^b^	1.5	196^a^	1.2	6.96 (2)	0.01**
Cyberviolence perpetration	3,134^a^	17.9	3,904^b^	23.0	2,634^c^	15.6	312.07 (2)	0.08***
(*n* = 51,348; young adolescent = 17,514, middle adolescent = 16,998, older adolescent = 16,836)								
Cyber verbal abuse	2,695^a^	15.4	3,630^b^	21.4	2,350^c^	14.0	372.63 (2)	0.09***
Cyber defamation	762^a^	4.4	994^b^	5.8	585^c^	3.5	112.12 (2)	0.05***
Cyberstalking	326^a^	1.9	421^b^	2.5	282^a^	1.7	30.44 (2)	0.02***
Online sexual abuse	239^a^	1.4	335^b^	2.0	251^a^	1.5	22.18 (2)	0.02***
Personal information leakage	332^a^	1.9	511^b^	3.0	382^c^	2.3	47.16 (2)	0.03***
Cyberbullying	400^a^	2.3	397^a^	2.3	280^b^	1.7	23.13 (2)	0.02***
Cyber extortion	197^a^	1.1	276^b^	1.6	205^a^	1.2	18.51 (2)	0.02***
Cyber coercion	244	1.4	276	1.6	225	1.3	5.50 (2)	0.01
Education is important	12,387^a^	95.7	11,337^b^	91.6	10,927^c^	90.4	278.75 (2)	0.09***
(*n* = 37,407; young adolescent = 12,949, middle adolescent = 12,374, older adolescent = 12,084)								
Education provided is helpful	11,322^a^	93.8	9,304^b^	83.6	8,106^c^	79.5	1019.49 (2)	0.18***
(*n* = 33,427; young adolescent = 12,069, middle adolescent = 11,168, older adolescent = 10,190)								
Aware of official help provided	9,542^a^	54.5	9,436^a^	55.5	7,753^b^	46.1	366.00 (2)	0.09***
(*N* = 56,306; young adolescent = 17,514, middle adolescent = 16,998, older adolescent = 16,836)								
Aware of legal consequences	10,126^a^	57.8	10,742^b^	63.2	10,186^c^	60.5	104.43 (2)	0.05***
(*n* = 51,348; young adolescent = 17,514, middle adolescent = 16,998, older adolescent = 16,836)								

*Note:* Subscripts (a, b, c) indicate significant differences.

*** *p* < 0.001.

** *p* < 0.05.

Among all of the surveyed adolescents, the middle adolescents were found to have experienced the most cyberviolence victimisation and perpetration: 20.8% victimisation and 23% perpetration versus 17.7% victimisation and 17.9% perpetration for the young adolescents and 14.6% victimisation and 15.6% perpetration for the older adolescents. These group differences were significant (victimisation: *χ* ^2^ = 221.42, Cramér's *V* = 0.07, *p* < 0.001; perpetration: *χ* ^2^ = 312.07, Cramér's *V* = 0.08, *p* < 0.001). With the exception of cyberbullying, which was reported most by the young adolescents, the middle adolescents reported having fallen prey to all types of cyberviolence significantly more than the other age groups. A similar trend was observed in cyberviolence perpetration; that is, the middle adolescents reported having engaged in most types of cyberviolence (verbal abuse, defamation, stalking, sexual abuse, leakage of personal information, and extortion in a digital setting) significantly more than the young and older adolescents. Furthermore, the young and middle adolescents reported similar levels of their own cyberbullying behaviour. The strength of these relationships was relatively weak (Cramér's *V* ranging from 0.01 to 0.09).

With increasing age, the adolescents seemed to find preventive education to be less important and less helpful. Specifically, 95.7% of the young adolescents, 91.6% of the middle adolescents, and 90.4% of the older adolescents perceived it as important and necessary (*χ* ^2^ = 278.75, Cramér's *V* = 0.09, *p* < 0.001), and 93.8% of the young adolescents, 83.6% of the middle adolescents, and 79.5% of the older adolescents perceived it as helpful (*χ* ^2^ = 1019.49, Cramér's *V* = 0.18, *p* < 0.001) in dealing with cyberviolence incidents. Notably, only slightly over half of the young (54.5%) and middle (55.5%) adolescents were aware of ways to ask for help when experiencing cyberviolence, and the percentage dropped to 46.1% for the older adolescents (*χ* ^2^ = 366.00, Cramér's *V* = 0.09, *p* < 0.001). In terms of the legal consequences of committing cyberviolence, significantly more of the middle adolescents (63.2%) reported such knowledge, compared with the older (60.5%) and young (57.8%) adolescents (*χ* ^2^ = 104.43, Cramér's *V* = 0.05, *p* < 0.001). The strength of association in these group differences was weak to moderate (0.05–0.18).

### 5.4 Yearly Prevalence of Cyberviolence Victimisation and Perpetration, 2017–2024

Table 3 shows the yearly breakdown of the adolescents' reported cyberviolence experiences. Among the entire sample, the adolescents surveyed in 2018 reported the highest incidence of victimisation (20.8%), followed by those surveyed in 2022 (20.3%) and 2020 (19.7%). The 2021 respondents reported the fewest cyberviolence victimisation incidents (13.7%). This difference was significant (*χ* ^2^ = 209.62, *p* < 0.001) but with a weak effect size (Cramér's *V* = 0.06). Breaking down the results by type of cyberviolence, cyberstalking (4.6%), digital sexual abuse (3.2%), and cyber coercion (2%) had the highest reported incidence rates in 2021, while the rate of online verbal abuse (18.7%) and cyberbullying (2.4%) were highest in 2018. Online defamatory incidents (6.6%) and cyber theft activities (2.4%) were most prevalent in 2020, and 2017 displayed the highest incidence rate of the online leakage of personal information (2.6%). In general, cyberviolence victimisation incidence rates, which began at 16.6% in 2017, peaked in 2018 at 20.8% and subsequently declined slowly over the following years (19% in 2019, 19.7% in 2020 and 13.7% in 2021) until it peaked again in 2022 at 20.3%, before declining to 18.2% in 2023 and 16.6% in 2024.

**TABLE 3:** Yearly prevalence of cyberviolence victimisation and perpetration, 2017–2024 (*N* = 56,306).

Characteristics	2017 (*n* = 4500)	2018 (*n* = 4662)	2019 (*n* = 4779)	2020 (*n* = 4779)	2021 (*n* = 9017)	2022 (*n* = 9693)	2023 (*n* = 9218)	2024 (*n* = 9479)
	*n* (%)	*n* (%)	*n* (%)	*n* (%)	*n* (%)	*n* (%)	*n* (%)	*n* (%)
Cyberviolence victimisation	745^a,b^ (16.6)	972^c^ (20.8)	907^b–d^ (19.0)	976^c,d^ (19.7)	1,231^e^ (13.7)	1,972^c^ (20.3)	1,681^a,b,d^ (18.2)	574^a^ (16.6)
(*χ* ^2^ = 209.62, df = 7, Cramer's *V* = 0.06)***								
Cyber verbal abuse	657^a,b^ (14.6)	870^c^ (18.7)	807^b–d^ (16.9)	804^b,d^ (16.2)	809^e^ (9.0)	1,713^c,d^ (17.7)	1,412^b^ (15.3)	1,261^a^ (13.3)
(*χ* ^2^ = 404.79, df = 7, Cramer's *V* = 0.09)***								
Cyber defamation	241^a–i^ (5.4)	286^f–i^ (6.1)	296^d,e,h,i^ (6.2)	325^c,e,g,i^ (6.6)	424^b^ (4.7)	624^a,c–i^ (6.4)	550^a,c–i^ (6.0)	557^a,c–i^ (5.9)
(*χ* ^2^ = 35.95, df = 7, Cramer's *V* = 0.03)***								
Cyberstalking	118^a^ (2.6)	155^a,b^ (3.3)	150^a,b^ (3.1)	196^b,c^ (4.0)	414^c^ (4.6)	339^a,b^ (3.5)	253^a^ (2.7)	266^a^ (2.8)
(*χ* ^2^ = 76.06, df = 7, Cramer's *V* = 0.04)***								
Online sexual abuse	87^a,b^ (1.9)	127^b,c^ (2.7)	106^a,b^ (2.2)	142^b,c^ (2.9)	289^c^ (3.2)	242^b,c^ (2.5)	204^a,b^ (2.2)	161^a^ (1.7)
(*χ* ^2^ = 57.82, df = 7, Cramer's *V* = 0.03)***								
Personal information leakage	115^a–c^ (2.6)	118^a–c^ (2.5)	99^c,d^ (2.1)	154^b^ (3.1)	183^a,c,d^ (2.0)	229^a–c^ (2.4)	178^a,c,d^ (1.9)	152^d^ (1.6)
(*χ* ^2^ = 45.78, df = 7, Cramer's *V* = 0.03)***								
Cyberbullying	68^a,b^ (1.5)	110^b,c^ (2.4)	81^a,b^ (1.7)	147^c^ (3.0)	192^b,c^ (2.1)	177^b^ (1.8)	157^b^ (1.7)	106^a^ (1.1)
(*χ* ^2^ = 77.29, df = 7, Cramer's *V* = 0.04) ***								
Cyber extortion		79^b–d^ (1.7)	47^d–f^ (1.0)	119^c^ (2.4)	173^b,c^ (1.9)	153^b,d,f^ (1.6)	98^e,f^ (1.1)	62^e^ (0.7)
(*χ* ^2^ = 183.27, df = 6, Cramer's *V* = 0.06) ***								
Cyber coercion		76^b–f^ (1.6)	54^e,f^ (1.1)	83^b–f^ (1.7)	178^d^ (2.0)	166^c,d,f^ (1.7)	111^b,c,e,f^ (1.2)	106^b,e^ (1.1)
(*χ* ^2^ = 108.99, df = 6, Cramer's *V* = 0.04) ***								
Cyberviolence perpetration	730^a^ (16.2)	970^b,c^ (20.8)	860^a,d^ (18.0)	469^e^ (9.5)	1,236^f^ (13.7)	1,999^b,c^ (20.6)	1,777^c,d^ (19.3)	2,100^b^ (22.2)
(*χ* ^2^ = 558.05, df = 7, Cramer's *V* = 0.10) ***								
Cyber verbal abuse	680^a^ (15.1)	901^b^ (19.3)	801^a,c^ (16.8)	446^d^ (9.0)	1,059^e^ (11.7)	1,858^b^ (19.2)	1,656^b,c^ (18.0)	1,720^b,c^ (18.1)
(*χ* ^2^ = 470.54, df = 7, Cramer's *V* = 0.09) ***								
Cyber defamation	129^a^	214^b–e^	173^a,d,e^	78^f^	355^c,e^	532^b^	435 ^b–e^	503^b^
	(2.9)	(4.6)	(3.6)	(1.6)	(3.9)	(5.5)	(4.7)	(5.3)
(*χ* ^2^ = 181.95, df = 7, Cramer's *V* = 0.06) ***								
Cyberstalking	63^a–e^ (1.4)	97^d–f^ (2.1)	73^c,e,f^ (1.5)	39^b^ (0.8)	206^f^ (2.3)	196^a,c–f^ (2.0)	195^a,c–f^ (2.1)	199^a,c–f^ (2.1)
(*χ* ^2^ = 55.72, df = 7, Cramer's *V* = 0.03) ***								
Online sexual abuse	46^a–d^ (1.0)	70^c–e^ (1.5)	49^b,d^ (1.0)	30^b^ (0.6)	159^e^ (1.8)	165^a,c,e^ (1.7)	170^e^ (1.8)	166^e^ (1.8)
(*χ* ^2^ = 58.63, df = 7, Cramer's *V* = 0.03) ***								
Personal information leakage	97^a,b^ (2.2)	102^a,b^ (2.2)	90^b^ (1.9)	39^c^ (0.8)	208^a,b^ (2.3)	230^a,b^ (2.4)	238^a,b^ (2.6)	260^a^ (2.7)
(*χ* ^2^ = 67.49, df = 7, Cramer's *V* = 0.04) ***								
Cyberbullying	69^a–e^ (1.5)	88^d–f^ (1.9)	87^c,e,f^ (1.8)	50^b^ (1.0)	199^a,c–f^ (2.2)	229^f^ (2.4)	196^a,c–f^ (2.1)	209^a,c–f^ (2.2)
(*χ* ^2^ = 42.17, df = 7, Cramer's *V* = 0.03) ***								
Cyber extortion		58^b,c^ (1.2)	47^b,c^ (1.0)	39^c^ (0.8)	145^b^ (1.6)	157^b^ (1.6)	137^b^ (1.5)	134^b^ (1.4)
(*χ* ^2^ = 92.70, df = 6, Cramer's *V* = 0.04) ***								
Cyber coercion		68^b,c^ (1.5)	50^c^ (1.0)	24^d^ (0.5)	138^b,c^ (1.5)	160^b,c^ (1.7)	136^b,c^ (1.5)	193^b^ (2.0)
(*χ* ^2^ = 134.94, df = 6, Cramer's V = 0.05)***								

*Note:* ***p < 0.001. Superscripts (a–i) indicate significant differences.

Over the study period, the highest percentage of adolescents reporting having engaged in cyberviolence perpetration was in 2024 (22.2%), followed by 2018 (20.8%) and 2022 (20.6%). Although this group difference was significant (*χ* ^2^ = 558.05, *p* < 0.001), the strength of this association was weak (Cramér's *V* = 0.10). In terms of the respondents' rates of perpetration according to type of cyberviolence, 2024 saw the highest incident rates of online leakage of personal information (2.7%), cyber coercion (2%), and online sexual abuse (1.8%, which was the same rate recorded in both 2021 and 2023), while 2022 had the highest rates of reported defamatory activities (5.5%), bullying episodes (2.4%), and extortion activities (1.6%, which was the same rate recorded in 2021). Incidents of cyberstalking (2.3%) and online sexual abuse (1.8%) were most prevalent in 2021, and 2018 recorded the highest perpetration rate of online verbal abuse (19.3%). In contrast to the trend of cyberviolence victimisation during this eight‐year period, the perpetration rate began at 16.2% in 2017, seemed to peak in 2018 at 20.8%, and subsequently dropped to the lowest point in 2020 at 9.5% before the rate climbed again, peaking in 2024 at 22.2%.

## 6 Discussion

This study offers insights into the prevalence of cyberviolence in South Korean adolescents, based on nationally representative data spanning an eight‐year period (2017–2024). To the best of the authors' knowledge, the period covered in this study was the longest and the sample was the largest to date for research of this kind, and it was the first study to observe the pre‐ and post‐COVID‐19 pandemic. By spanning the COVID‐19 years (2020–2022) as well as the years immediately before and after, the study captured unique shifts in online behaviour, showing that, contrary to most Asian studies' findings, cyberviolence incidence rates fell during the pandemic in South Korea. The most salient findings were as follows. The female adolescents and adolescents aged 16–18 years reported having spent more time on the Internet (by three hours or more daily) than their male and younger counterparts. In terms of cyberviolence perception, more of the female adolescents and adolescents aged 16–18 years were also able to recognise different types of cyberviolence as problematic than their male and younger counterparts. The 12‐month prevalence rates of cyberviolence victimisation and perpetration during the study period were 17.9% and 18%, respectively, with a higher prevalence among males than females (20.7% vs. 14.8% for victimisation and 22.5% vs. 13.2% for perpetration). Nonetheless, only 9% of the surveyed adolescents had witnessed some sort of cyberviolence during the 12 months prior to being surveyed. Most of the respondents (89.9%) were capable of recognising cyberviolence as problematic, which is perhaps explained by the high percentage of them who viewed preventive education as an important (92.6%) and helpful (86.1%) element in online safety. However, not many of them were aware of the official support available for victims of cyberviolence (52.2%) or the legal consequences of engaging in cyberviolence (63.3%).

Several noteworthy trends and experiences among South Korean adolescents with respect to cyberviolence have emerged that warrant further discussion. First, relative to the female adolescents, the male adolescents were generally more likely to experience both victimisation and perpetration of cyberviolence across most types of such violence, the one exception being that the female adolescents were significantly more likely than their male counterparts to fall prey to cyberstalking. Although these findings are in line with many recent empirical studies on cyberviolence, particularly on cyberbullying (e.g., Chan and Wong 2019; Choi et al. 2022), cyberstalking (e.g., Maran and Begotti 2019; Pereira and Matos 2016), and online sexual abuse (e.g., Jonsson et al. 2019; Sklenarova et al. 2018), certain studies have failed to find any significant differences between the sexes in this context (e.g., Li 2007; Pedersen et al. 2023; Slonje and Smith 2008; Toomey and Russell 2016). This inconclusive evidence of differences in cyberviolence victimisation and perpetration according to sex may be due in part to the fact that female adolescents, who tend to be more submissive than male adolescents in face‐to‐face settings (e.g., in‐person bullying and assaultive behaviour; Chan 2021), may be less inhibited when they can enjoy the anonymity and other equalising characteristics of cyberspace, and this empowerment renders them more willing to engage in cyberviolence (Ybarra and Mitchell 2004). In contrast, another possibility is that female adolescents may be more reluctant than male adolescents to retaliate against their perpetrators through mediums by which they themselves have been victimised (Chan and Wong 2017). For instance, Chan and Wong (2020) found that male adolescents who were once victims of cyberbullying were more likely than their female counterparts to engage in cyberbullying (i.e., victim‐offenders; 16.7% of males vs. 12.8% of females).

In terms of age, the surveyed adolescents between 13 and 15 years of age (i.e., middle adolescents) reported a higher prevalence of both cyberviolence victimisation and perpetration than the young (10–12 years old) and older (16–18 years old) adolescents across most types of cyberviolence, the sole exception being that cyberbullying victimisation was more prevalent among the young adolescents. Consistent with the literature on most types of cyberviolence (e.g., cyberbullying and cyberstalking), the adolescents aged 12–15 years tended to report more experiences with cyberviolence as both victims and perpetrators than the younger and older adolescents (e.g., Novo et al. 2014 [general cyberviolence]; Slonje and Smith 2008 [cyberbullying]; Walrave and Heirman 2011 [cyberbullying]; Pereira and Matos 2016 [cyberstalking]). However, the present study's findings conflicted with the findings of previous studies that cyberbully victimisation was prevalent among younger adolescents (e.g., under 12 years; Patchin and Hinduja 2022; Vismara et al. 2022) and that sexual victimisation in the cyberworld tended to occur in older adolescents (16–17 years; Montiel et al. 2016). Particularly with cyberbullying victimisation, younger age groups were increasingly affected, partly due to the growing accessibility and frequent use of the Internet in recent years, especially during and after the COVID‐19 pandemic.

The COVID‐19 pandemic impacted the prevalence of cyberviolence, as the associated social isolation regulations limited face‐to‐face interaction, leading to a significant rise in the use of the Internet, social media, and online activities between 2020 and 2022. A higher probability of experiencing all types of cyberviolence was the expected result of this phenomenon (Shin and Choi 2021). Many empirical studies, especially those surveying Asian (e.g., Chinese, Malaysian, Vietnamese) and Australian samples, have reported an increased prevalence of cyberviolence during the COVID‐19 pandemic compared with previous years (see Huang et al. 2024; Sorrentino et al. 2023 for a review). However, studies conducted in Western countries (e.g., Canada, the U.S.) have revealed the opposite trend; that is, a decrease in cyberviolence rates. Inconsistent with most studies that sampled Asian populations but generally in line with Shin and Choi's (2021) study of South Korean adolescents, incidents of cyberviolence victimisation and perpetration during the pandemic were reported in the present study to be generally lower than in the pre‐ (2017–2019) and post‐pandemic (2023–2024) years. In fact, 2021 and 2020 recorded the lowest incidence rates of cyberviolence victimisation (13.7%) and perpetration (9.5%), respectively. In South Korea, the decrease in cyberviolence incidents could be explained by the increase in supervised time spent on the Internet during the COVID‐19 pandemic. Specifically, pandemic prevention measures required school administrators and teachers to supervise and monitor the safety and health of their students and school employees, which may have increased their awareness and responsiveness to individual, socio‐emotional wellbeing, especially in the context of cyberspace (Azizi et al. 2021; Bacher‐Hicks et al. 2022). Such strategies may have reduced incidents of cyberviolence (e.g., cyberbullying, leakage of personal information, cyber extortion). Moreover, social distancing strategies may have reduced traditional, in‐person (physical) bullying, which has a strong connection with cyberbullying (Chan and Wong 2019; Estevéz et al. 2020). Furthermore, the lockdown measures taken during the pandemic allowed most people to spend more time with family, thus providing more access to family support to counter or prevent negative online experiences (Magis‐Weinberg et al. 2021).

Caution should be exercised in interpreting the findings of this study, owing to several limitations. First, the inherent limitations of the data collected should be acknowledged. While most of the annual, national surveys examined in this study were administered in the field—specifically, in physical classrooms—the 2017 to 2019 surveys were administered by postal mail, while the 2020 survey was administered online, due to the restrictions imposed during the COVID‐19 pandemic. Furthermore, the data collected from 2017 to 2020 were collected before the survey was designated as informing official national statistics. As a result, methodological differences exist in the survey design, data collection, and analysis between these and later surveys. Such methodological differences may limit the reliability of comparisons between the surveys conducted in these eras. Another limitation is the use of self‐reported data, which may introduce response biases affecting internal validity. Despite ongoing debates about the veracity of information from self‐report methods (Schwarz 1999), self‐reported data are neither inherently valid nor invalid; rather, they vary with the methodological sophistication of the data collection (Del Boca and Noll 2002). The data examined in this study are cross‐sectional in nature and not panel data; hence, no temporal relationship among the surveyed adolescents can be inferred. To strengthen the methodology, future researchers on this topic should consider adopting a single data collection method (either in‐person or online surveys) and an experimental approach to establish stronger causal inferences. Nonetheless, as this national survey on cyberviolence has been conducted annually since 2013 and focus groups of students and teachers have been carried out to pilot test the questionnaire items and the procedures of the survey (E. Lee et al. 2024a), the minimum validity of the data is assured.

## 7 Conclusions

The advent of social media, increasing use of text messaging as a primary communication method, and extensive marketing of new communication and Internet technologies have engendered a world of constant change, especially among those younger than 18 years. The current generation of adolescents has grown up in a technology‐based society in which the Internet and cyberspace are taken for granted, online relationships develop quickly, personal information is shared, and harassment remains a significant risk. As a result, the need for Internet safety and awareness appears to be growing exponentially, especially since the COVID‐19 pandemic.

The present study, which drew on the largest sample of adolescent cyberviolence data available in South Korea over the longest study period to date, provides a robust evidence base to inform national and international cyberviolence prevention and intervention policy. Furthermore, it provides the strongest evidence to date of sex, age, and temporal patterns in cyberviolence among South Korean adolescents and offers a model for other high‐connectivity countries to track and respond to online harms. Its findings offer certain implications. e.g., preventive education is deemed to be important and helpful in addressing cyberviolence issues, whereas adolescent awareness of available support for those coping with victimisation and of the legal consequences of engaging in cyberviolence in South Korea remains relatively low. Hence, more work needs to be done in this area.

Adolescents and parents should be informed of Internet dangers through the media and schools, including, ironically, via the Internet itself. Preventive and intervention efforts must be efficiently strategised and multipronged. The supervision and management of cyber environments should be further strengthened. Governments may develop injunctive and descriptive norms of cyberviolence perpetration and enhance online education programmes to increase the public's moral engagement in and awareness of cyberviolence issues (e.g., legal consequences) and support resources (e.g., helplines, victim services) (Huang et al. 2024). Furthermore, governments and other official bodies (e.g., UNESCO) should collaborate with online platforms to use artificial intelligence tools to monitor and clear disinformation, illegitimate and extremist online content, and hate speech on various social media and Internet platforms (Kee et al. 2022).

At the school level, key stakeholders in the education system, such as school administrators and teachers, play an important role in addressing cyberviolence problems. Creating a secure and pleasant school climate could foster an atmosphere conducive to a constructive learning environment for adolescents. Training in personal development skills to promote prosocial functioning (e.g., unselfishness, resilience) and regulation of emotions (e.g., victim empathy, anger management) could prevent negative transactions that perpetuate both online and offline violent behaviours (Chan and Wong 2019). Through such training and with their teachers' support, adolescents should be able to develop empathy and a sense of respect for others, thus decreasing their involvement in cyberviolence.

Parents and caregivers, for their part, remain an important agency for the continuing socialisation of adolescents. Studies have consistently noted the importance of parental involvement in the life of adolescents in affecting their future involvement in delinquency, both as victims and perpetrators (e.g., Chan 2019; Craig 2015; Hoeve et al. 2012). Parents and caregivers must recognise unhealthy or harmful online activities and address them immediately with clear rules and expectations; this can be effective in preventing and intervening in risky online behaviour. A secure and healthy (i.e., responsive and supportive) parent–child relationship is a key factor in protecting adolescents against delinquency and other problematic behaviours (e.g., alcohol and drug use). It is undisputed that a positive family atmosphere, effective parental monitoring, better communication within the family, and overall positive school experiences and involvement are of the utmost importance in fostering adolescents' healthy psychosocial functioning, making them less likely to engage in or fall victim to cyberviolence.

Notwithstanding the noted data limitations, this study is important in two major ways. First, it advances our knowledge about the prevalence, nature, and perception of adolescent cyberviolence in South Korea. Second, it extends our understanding of the differences in adolescents' perceptions and experiences of cyberviolence between the sexes and various age groups. It should also be noted that the dataset used provided the best available and nationally representative data to study cyberviolence in South Korea. As such, this study provides solid groundwork for additional research to better characterise the nature of, trends in, and dynamics of various types of cyberviolence among adolescents in South Korea.

This study presents, for the first time, how preventive education, awareness of support services, and knowledge of legal consequences vary with age and sex over time in South Korean adolescents. It further establishes a new baseline for adolescent cyberviolence trends in one of the world's most highly connected countries, shows unexpected COVID‐era declines in incidents, and documents significant sex and age disparities and prevention awareness gaps, offering an evidence base for future interventions and policy. It is essential to continue building our knowledge about this offending behaviour online, to garner further important implications for practices in prevention and intervention strategies.

## Author Contributions

**Heng Choon (Oliver) Chan:** conceptualization, methodology, investigation, formal analysis, data curation, writing – original draft, review and editing. **Hong‐Jun Park:** data acquisition, writing – review and editing. **Evelyn Svingen:** writing – review and editing.

## Funding

The authors have nothing to report.

## Ethics Statement

This was a secondary data analysis. No ethical approval was required.

## Conflicts of Interest

The authors declare no conflicts of interest.

## Data Availability

The data that support the findings of this study are available on request from the corresponding author.
